# Laparoscopic Splenectomy in a Child with Moyamoya Syndrome, Hereditary Spherocytosis, and Interstitial Lung Disease: A Mere Coincidence or Partnership Based on Genetic Similarities

**DOI:** 10.1155/2011/253434

**Published:** 2011-12-29

**Authors:** Kasra Karvandian, Zahid Hussain Khan, Jayran Zebardast, Sayed Rohollah Miri

**Affiliations:** ^1^Department of Anesthesiology, Tehran University of Medical Sciences, Tehran 1441963811, Iran; ^2^Deputy of research Imam Khomeini Hospital, Tehran University of Medical Sciences, Tehran, Iran; ^3^Tehran University of Medical Sciences, Tehran, Iran

## Abstract

A case of moyamoya syndrome and spherocytosis with concurrent interstitial lung disease who underwent laparoscopic splenectomy is being reported. A theory regarding their coexistence is being forwarded together with their anesthetic management. According to our search, this is the fourth case of moyamoya syndrome and the first case with an associated interstitial lung disease in a 10-year-old child.

## 1. Introduction

The disease moyamoya evolves from the Japanese word *moya* meaning haziness resembling an exhaled cigarette smoke puffy angiographic appearance of the collateral cerebral vasculature distant to the stenotic and occluded prominent vessels around the circle of Willis [1], first described in the literature by Suzuk and Takaku [2].

To our knowledge, so far, only three pediatric cases of moyamoya syndrome have been reported. Owing to the paucity of the literature regarding an association of moyamoya disease and hereditary spherocytosis, we are reporting another case of moyamoya syndrome with hereditary spherocytosis causing splenomegaly and an interstitial lung disease (ILD), (Figure 3) as a result of hemosiderosis following repeated blood transfusions in treating chronic anemia. This is the first case who had an additional finding of ILD and who underwent a laparoscopic splenectomy. We would be dwelling on the probable theories of their coexistence on the basis of pathogenesis and finally introducing our own model of anesthetic management of this solitary case in the literature.

## 2. Case Description

**Figure 1:**
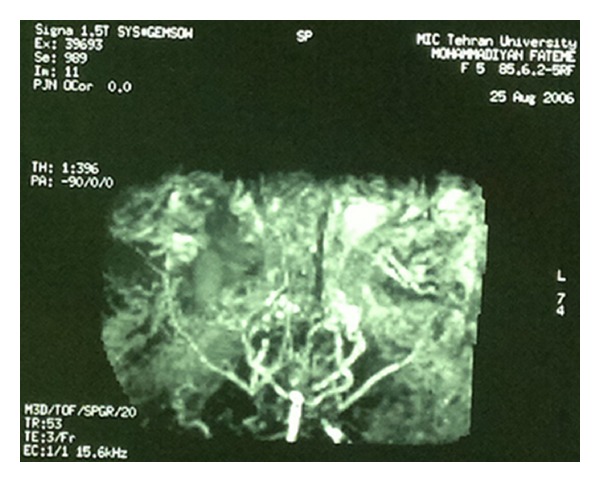
Stenosis of both internal carotid arteries and collateral network signifying moyamoya appearance.

**Figure 2:**
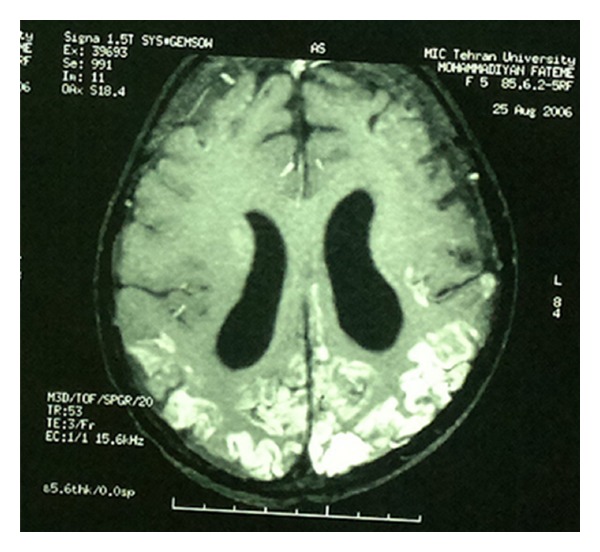
Hypertrophy of leptomeningeal arteries with retrograde cortical feeding.

A 10-year-old girl diagnosed as a case of hereditary spherocytosis at the age of 1 year had an episode of convulsion at the age of 5 years following which she developed slurred, incoherent, and incomprehensible speech, myoclonic movements, and left sided hemiparesis. Following her hemorrhagic stroke, she had been receiving aspirin and warfarin. During the course of her illness, she had been receiving blood transfusion off and on as a result of decrement in hemoglobin levels. An osmotic fragility test had been performed by placing the patient's red blood cells in a saline solution for 24 hours. Compared to normal cells, the patient's red blood cells had burst sooner, establishing the diagnosis of hereditary spherocytosis once again prior to undertaking the scheduled laparoscopic splenectomy. A cerebral angiography (Figure 1) depicts the classical appearance of moyamoya disease as had been narrated in the past, with hypertrophied meningeal arteries reflecting retrograde feeding (Figure 2).

**Figure 3:**
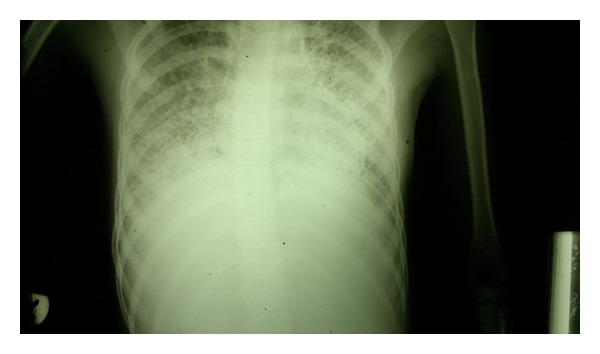
A chest radiograph showing interstitial lung disease.

The patient was scheduled for laparoscopic splenectomy. On general examination, the patient exhibited bizarre involuntary movements of the body especially evident in the cervicoscapular regions and the limbs coupled with facial grimaces, undefined smiles, and behavior reflecting gross mental retardation. The chest X-ray and CT scan favored an acute lung injury pattern (Figure 3) although the oxygen saturation on breathing room air was 97%. On auscultation, the lungs were clear and an echocardiographic report of the heart had been normal.

After adjusting routine monitors, the patient received preinduction fentanyl and midazolam in 50 micg and 1 mg dosages, respectively. Induction was performed with thiopental sodium 5 mg/kg, and a noncuffed 6.5 size ID endotracheal tube was introduced after atracurium 0.5 mg/kg. Remifentanil and propofol infusions were used for maintenance of anesthesia. During the operation, phenobarbital 75 mg and hydrocortisone 30 mg were given. The patient received 2 L/min oxygen during the operation which lasted 3 hours. The end tidal carbon-dioxide level was maintained within the range of 35–40 mmHg. The anesthetic course was uneventful, and the patient was extubated and transferred to the ward after a 30 min followup in the postanesthesia care unit. Penicillin G 100,000 units/kg was started postoperatively and repeated every 4 h. On the 5th postoperatively day, the patient was discharged and asked to report for followups.

## 3. Discussion

Three cases of moyamoya syndrome and its association with hereditary spherocytosis have so far been reported [3–5]. Since our case is the fourth one with such an association, the foremost question that arises in one's mind is if this association of moyamoya disease and hereditary spherocytosis could be an incidental finding or this coincidence could be explainable on definite physiopathological grounds. Since four cases of such an association have so far been introduced, this association could not perhaps be a mere coincidence, but in all probability, the hereditary spherocytosis could perhaps have contributed to the genesis of moyamoya disease on the assumption that chronic hypoxia secondary to hemolysis of the spherocytes could be acting as a precipitating cause of oxygen decrement in the brain of these patients with a possible genetic predisposition, thus leading to sprouting of collaterals to compensate for oxygen deprivation. A hypothesis somehow resembling this one had been expressed in the past [6], but lack of evidence failed to establish it. Further epidemiological reports could perhaps establish the cause and effect relationship. Our case had an added finding of ILD not so far reported in the literature. As most of these patients undergo splenectomy at around 5 yr of age, ILD has never been reported. This patient underwent splenectomy at a later age because of parent's refusal for surgery, and repeated blood transfusions during this time lag could most probably have led to hemosiderosis and ILD.

In our patient, a laparoscopic splenectomy was performed where in the intra-abdominal pressure was maintained around 10–12 mmHg. The carbon-dioxide insufflation was perhaps helpful, as the end tidal PaCO_2_ could be maintained above 30 mmHg. Hypocapnia is preferably avoided in patients with moyamoya disease, because the ensuring cerebral vasoconstriction could further decrease the already compromised cerebral blood flow. In our anesthetic management, we maintained adequate hydration of the patient presuming that dehydration would trigger the process of stasis and its attendant complications.

Halothane has been used with success [7] owing to the long-held assumption that halothane produces lesser amount of coughing and breath holding [8], but the fact is that it does produce them and that little amount in itself could be deleterious for a patient with moyamoya disease whose cerebrovascular compliance is already compromised, thus making these patients especially vulnerable to developing intraoperative ischemic episodes. Again, Suriano et al. [7] chose isoflurane as part of their maintenance anesthesia on the grounds that it had cerebral protective effects during transient cerebral ischemia in adults undergoing carotid endarterectomy [9]. We feel that the findings attributed to isoflurane regarding cerebral protection during transient cerebral ischemia in patients undergoing carotid end-arterectomy cannot be extrapolated to children having moyamoya disease, because the pathogenesis is different with moyamoya disease having already ushered in a host of changes in the intracranial arteries in the form of stenosis, total blockade, and compensation in the form of collaterals. Under such circumstances, it would not be prudent to choose isoflurane, because like the other halogenated agents, it also can abolish autoregulation in a dose-dependent manner besides causing a reduction in peripheral vascular resistance and cerebrovascular tone. Cerebral autoregulation is already disturbed in this subset of patients, and any further disturbance in it could hasten the process of ischemia that already exists, unlike carotid endarterectomy, where ischemia is transiently produced in a patient in the face of normal autoregulation. We avoided halogenated agents to prevent vasodilatation in the normal intracranial vasculature ushering in a steal phenomenon and thus jeopardizing the regional cerebral blood flow that could in itself cause an intraoperative or a postoperative infarct. The vicious cycle could also increase the cerebral blood volume, and thus raise an unnoticed raised intracranial pressure culminating in delayed awakening or nonawakening. We employed propofol and remifentanil for maintenance of anesthesia titrated to the needs of the patient.

While reviewing the medical records of children with moyamoya disease as reported by Soriano et al. [7], anesthesia had been induced by an inhalation of a mixture of halothane in 66% nitrous oxide and 33% oxygen. In our opinion, an inhalational induction in children with a mean age of 7.9 yr is fraught with stakes as coughing, crying, and breath holding can all lead to or else precipitate ischemic events in the already perilous neurovascular anatomy, where the normal collateral vessels are already maximally dilated and autoregulatory responses are absent or hampered. The blood pressure in our patient was maintained close to the baseline values on the grounds that deliberate or else inadvertent dangerous declines in blood pressure can cause a cerebral infarction [10]. Thus, adequate hydration and correct replacement of blood loss should be our goal in order to prevent any untoward complications that may come in the wake of these changes especially so as most of these children present with low preoperative hemoglobin levels.

As only few cases have been reported, a logical conclusion cannot be drawn regarding the modus operandi for anesthetic management but apparently owing to the fragile and vulnerable neurovascular pathological pattern in moyamoya disease and the concurrent presence of hereditary spherocytosis, it appears that we use anesthetic drugs that incur the least morbidity and mortality in this subset of population and optimize hydration and hemoglobin levels during the perioperative period.
